# Caught in the act: structural dynamics of replication origin activation and fork progression

**DOI:** 10.1042/BST20190998

**Published:** 2020-05-05

**Authors:** Jacob S. Lewis, Alessandro Costa

**Affiliations:** Macromolecular Machines Laboratory, The Francis Crick Institute, London NW1 1AT, U.K.

**Keywords:** biochemistry, cryo-electron microscopy, DNA replication, *in silico* reconstitution, single molecule

## Abstract

This review discusses recent advances in single-particle cryo-EM and single-molecule approaches used to visualise eukaryotic DNA replication reactions reconstituted *in vitro*. We comment on the new challenges facing structural biologists, as they turn to describing the dynamic cascade of events that lead to replication origin activation and fork progression.

## Introduction

**Figure 1.**
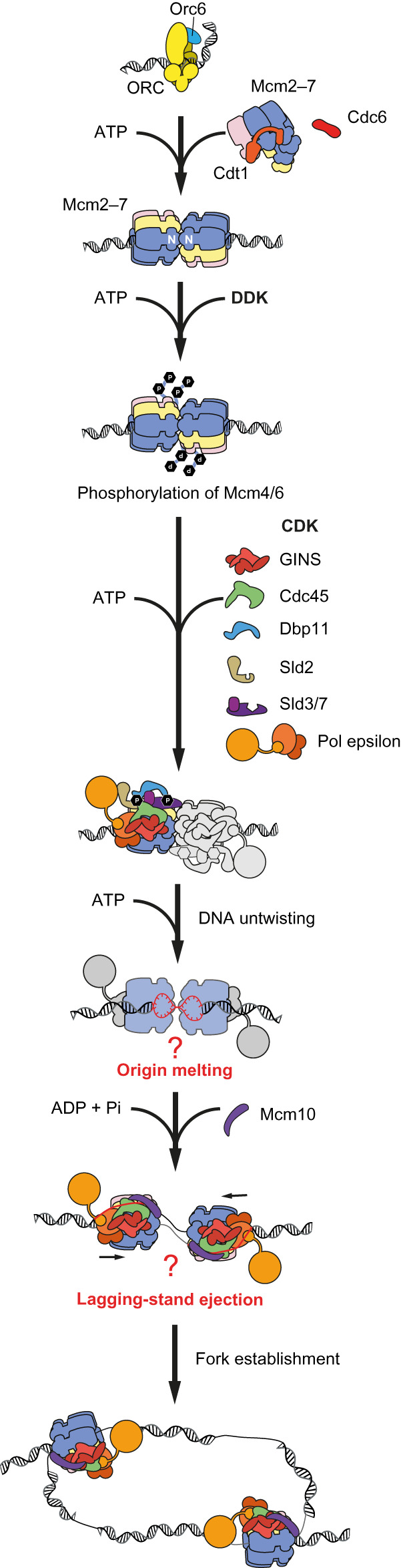
Licensing and activation of an eukaryotic origin of replication. Origin licensing is the recruitment of a set of two MCM ring-shaped helicases forming a double hexamer around duplex DNA. Origin DNA untwisting (and possibly melting) requires the recruitment of GINS and Cdc45 to MCM, which disrupt the double hexamer leading to the formation of two CMG assemblies, in an ATP-binding-dependent manner. Replication fork establishment requires of Mcm10 that switches on the ATP-hydrolysis function of CMG, causing lagging-strand ejection via an unknown mechanism.

Accurate transfer of genetic information from parental to daughter cells requires that chromosome replication is finely tuned, so that DNA is faithfully copied only once per cell cycle [1]. Failures in this process can lead to cellular abnormalities, genetic disease and cancer. *In vitro* reconstitution studies using budding yeast proteins revealed that the replication machinery is assembled in three temporally separated steps (Figure 1) [2–4]. First, the MCM helicase is loaded onto replication start sites (origins), during a process known as licensing that occurs in the G1 phase of the cell cycle. Here, the Origin Recognition Complex (ORC) associates with Cdc6 and recruits a set of two hexameric MCM rings, initially associated with the loading factor, Cdt1 [5,6]. Helicase loading requires ATP hydrolysis by MCM [7,8], which prompts the formation of a double hexameric MCM ring encircling duplex DNA [4,9–11]. A second step involves untwisting of the double helix [3], which is promoted by the association of Cdc45 and GINS to the MCM, together forming the CMG holo-helicase [12,13]. The third step in origin activation requires the recruitment of the firing factor, Mcm10, which activates the ATPase powered DNA translocation function of CMG [3,14,15]. At this stage single-stranded DNA becomes exposed and serves as a template for the replicative polymerases, which dynamically associate with the replisome during fork progression [3,16]. Here, we review recent biochemical, single-molecule and structural studies on *in vitro* reconstituted reactions that recapitulate DNA replication at cellular rates. We comment on how integrative single-molecule and cryo-electron microscopy (cryo-EM) approaches can provide unprecedented insights into the molecular mechanisms of DNA replication.

## A sequential, quasi-symmetric mechanism for MCM helicase loading

**Figure 2.**
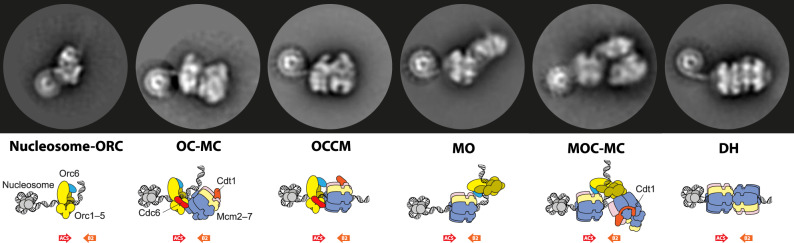
MCM double-hexamer formation follows a sequential, quasi-symmetric mechanism. A first ORC origin-association event involves an ACS, high-affinity DNA site. ORC binds and bends DNA, to allow for the recruitment of a first MCM ring, through a C-terminal MCM interaction. Upon release of ORC from the ACS site, a second ORC-binding event involves the N-terminal MCM domain and an inverted lower-affinity ORC-binding site. In this configuration, ORC is competent for recruiting a second MCM ring, following the same mechanism as the loading of the first MCM ring. The end result of the helicase loading reaction is the formation of a head-to-head double hexameric ring. The use of the ReconSil *in silico* reconstitution approach allows for the visualisation of a nucleosome, which flanks the ACS site in the reconstituted system and in yeast origins of replication.

The loading of a head-to-head MCM double hexamer establishes the symmetry required for bidirectional DNA replication. How the double hexamer is formed around duplex DNA has been the subject of intense debate. Both biochemical and structural work have shown that ORC binds and bends the DNA [17,18]. Upon association with Cdc6, ORC recruits one hexameric helicase ring via a set of C-terminal winged-helix domains [5,6,19]. The MCM ring contains a discontinuity (gate) between two subunits in the hexamer, which is kept open by the loading factor, Cdt1 [20,21]. Bent DNA is threaded through the MCM gate, leading to the formation of a short-lived ORC–Cdc6–Cdt1–MCM (OCCM) complex [19,22], which can be stabilised in the presence of a slowly hydrolysable ATP analogue. Several models have been proposed to explain the downstream molecular events that lead to MCM double-hexamer formation. Single-molecule work performed by the Bell group supports a sequential model whereby DNA loading of the first MCM ring drives origin-association of the second MCM ring. In the experimental conditions used, a single ORC complex is sufficient to drive double-hexamer formation. This observation suggests that the second helicase ring is recruited by the first loaded MCM, in particular via its N-terminal dimerisation interface [23,24]. Biochemical work from the Diffley group, however, indicates that the same winged-helix C-terminal elements in MCM are required for the loading of the first and second MCM rings, suggesting that both rings are recruited via the same OCCM mechanism [5]. Furthermore, efficient helicase loading requires two distinct ORC-binding events at inverted DNA sites, supporting a symmetric mechanism for double-hexamer formation [25]. While the two studies initially appeared to describe two distinct helicase-loading pathways, recent electron microscopy experiments indicate otherwise [22]. Time-resolved cryo-EM imaging revealed, in fact, that ORC first binds a high-affinity site (the ‘ACS’ element) on the origin of replication and recruits a first helicase ring by forming the OCCM (ORC–Cdc6–Cdt1–MCM) intermediate (Figure 2). Upon Cdc6 and Cdt1 release, as well as MCM ring closure, ORC disengages from the C-terminal domain of MCM. A second ORC-binding event occurs downstream on origin DNA, concomitantly engaging an inverted DNA site (the lower-affinity ‘B2’ element) and a previously unknown protein-binding site on the N-terminal face of MCM [22]. This new structural intermediate is named MO (MCM–ORC). The TFIIB-like ORC6 subunit [26] mediates the interaction and selectively recognises the MCM ring when this is locked around DNA. This mechanism ensures that recruitment of the second helicase occurs with a defined geometry, and only after the loading of the first MCM ring is complete [22]. In this inverted configuration, ORC is suitably positioned to recruit a second MCM ring via the OCCM pathway, eventually leading to double-hexamer formation. The cascade of events observed was completely unexpected, yet the observed structural transitions reconcile what initially appeared as contrasting models. As indicated by the Bell study, loading of the first MCM ring does drive loading of the second ring, and the N-terminal face of the first loaded MCM is indeed important in this process, as it mediates the second ORC-binding event [22,23]. Likewise, in accord with data from the Diffley group, both MCM rings are loaded via the same OCCM mechanism and involve two ORC-binding events that load two MCM rings in an inverted configuration [5,22,25]. Open questions remain — for example: does the same ORC complex always mediate the loading of both the first and second MCM ring without ever being released into solution? Or is loading mediated by two distinct ORC complexes, when working at physiological protein concentration? To prevent re-replication, origin licensing is inhibited by the CDK kinase which phosphorylates specific subunits in ORC [27–29]. What steps in the helicase-loading reaction are blocked by CDK to impair double-hexamer formation? Is it OCCM formation, MO assembly or perhaps downstream double-hexamer engagement? Hybrid biochemical, structural and single-molecule approaches will be needed to address these questions.

## Origin DNA melting and replication fork establishment

While helicase loading requires ATP hydrolysis by MCM, the double hexamer remains ADP-bound and does not unwind DNA [3,10,11]. Helicase activation requires the kinase function of both DDK and CDK. In particular, DDK phosphorylates MCM allowing for the recruitment of Sld3 and Cdc45 [30], while CDK phosphorylates Sld2 and Sld3, leading to the formation of a super-complex of firing factors (phospho-Sld3–Dpb11­–phospho-Sld2–GINS–Pol ε) [31,32], eventually promoting stable MCM engagement of helicase activators GINS and Cdc45 [2]. CMG assembly disrupts the double hexamer resulting in the formation of two holo-helicase particles. This process requires the release of ADP and binding of ATP by MCM, followed by the concomitant untwisting of origin DNA by 0.7 turns of DNA per CMG particle [3] (Figure 1). The structural state of untwisted origin DNA engaged by CMG remains unknown. In particular, it is unclear whether untwisting of 0.7 turns of the double helix can disrupt Watson–Crick base-pairing within the MCM cavity, which would nucleate origin DNA melting. Cryo-EM imaging of CMG assembled onto origin DNA is required to address this question.

ATP–CMG formation at origins fails to recruit the single-stranded binding protein RPA, indicating that additional components are required to establish a bidirectional replication fork [3]. The Mcm10 firing factor plays a key, yet elusive role in this context. Mcm10 promotes the ejection of the lagging-strand template from the MCM central channel and activates the ATP-hydrolysis function of the CMG, which powers single-stranded DNA translocation and DNA fork unwinding [3,14,15]. Two-dimensional single-particle EM analysis of CMG activated by Mcm10 demonstrated that the helicase advances with the N-terminal face forming the leading edge of the advancing replisome [3]. This observation, supported by 3D cryo-EM experiments [33–36], implies that the two helicases need to cross paths in order for bidirectional replication forks to be established. In this process, the strand ejected by one CMG helicase becomes the translocation strand of the second helicase (Figure 1). Several key questions remain unanswered. For example, is Mcm10 promoting ejection of the lagging-strand template from the MCM ring, and is this the trigger that activates the ATP-hydrolysis function in the CMG? Alternatively, is lagging-strand ejection a consequence of ATP-hydrolysis-driven leading-strand translocation along the leading-strand template, as proposed by a recent cryo-EM study on CMG fork translocation [33]? Do the Mcm10-activated, converging CMG particles exchange DNA strands as they pass one another? Or does strand ejection occur before the helicases cross paths? Is a specific gate used for strand ejection [16] as observed for the duplex DNA entry into MCM–Cdt1 during origin loading [20,21,37,38]? Addressing these questions will be important to establish whether CMG can actively translocate along duplex and not only single-stranded DNA, which is a compelling frontier question raised by a recent single-molecule study [16].

## Architecture and functions of the eukaryotic replisome

**Figure 3.**
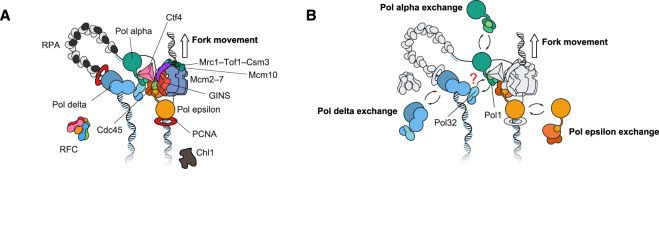
Architecture of the eukaryotic replication fork. (**A**) The CMG forms the organising centre of the eukaryotic replisome. Pol ε is positioned at the back of the advancing MCM helicase motor, directly interacting with the ATPase domain. Pol α is linked to the CMG via the homo-trimeric Ctf4 factor. Pol δ has been reported to directly interact with Pol α. Additional replisome factors mediate fast and efficient replication and other functions such as sister chromatid cohesion establishment and parental histone redeposition. (**B**) All three eukaryotic replicative polymerases can exchange into the replisome using a multi-site competitive exchange mechanism.

Not only does the CMG unwind the established DNA fork, it also serves as the organising centre of the replisome bringing together multiple DNA replication, genome and epigenome maintenance functions (reviewed in [39,40], Figure 3A). For example, the leading-strand polymerase Pol ε contacts both GINS and the ATPase (rear) face of the advancing MCM through a set of essential, non-catalytic modules [34]. Two functional elements are in turn flexibly tethered to the non-enzymatic core of Pol ε. One is the catalytic domain, which is likely free to engage and disengage the primer-template junction on the leading strand, hence allowing substrate access to the RFC clamp loader or other DNA polymerases [41]. The second flexible Pol ε element is the Dpb3–4 histone-like dimer [42], which plays a key role in re-depositing parental histones onto the leading-strand DNA [43,44]. Fork protection factors Csm3 and Tof1 are positioned ahead of the helicase, and they contact and stabilise duplex DNA as it enters the helicase pore [33]. These factors, together with Mrc1, support DNA replication at cellular rates in the *in vitro* reconstituted system; however, the mechanism is unknown [45,46]. Engagement of the incoming parental DNA at the fork by Csm3–Tof1 might directly increase the efficiency of replisome progression, as proposed in a recent cryo-EM study [33]. Also positioned at the leading edge of the replication fork is the homo-trimeric Ctf4 adaptor protein, linking the CMG helicase to a set of various client proteins. One example is Pol α, which primes Okazaki fragments on the lagging strand [47–49], and another is the helicase/nuclease Dna2, which functions during Okazaki fragment maturation [50]. Surprisingly, despite the direct interaction with lagging-strand enzymes, *in vitro* reconstitution experiments have failed to identify any role for Ctf4 in DNA replication *per se* [2,45], indicating that Ctf4 might play a more important role in critical chromosome maintenance functions. One of these roles is parental histone reshuffling at the replication fork. Indeed, Ctf4, alongside histone chaperone elements found in N-terminal Mcm2 and the Pol1 subunit of Pol α, have been found to function in the selective redeposition of parental histones onto lagging-strand DNA [51–55]. A second key activity is sister chromatid cohesion establishment, which indeed is the first function attributed to Ctf4 in cells [56]. The monomeric helicase Chl1, another known cohesion establishment factor, employs the same molecular mechanism to contact Ctf4, as identified for GINS and Pol α. Consistent with this, when point mutations were introduced in Chl1, designed to impair Ctf4 engagement, a sister chromatid cohesion establishment defect was identified, which nearly phenocopied the Ctf4 knockout [57]. Ctf4 can also bridge between two GINS (and therefore two CMG) assemblies, although the functional significance of this complex is unknown [47,58]. While it is established that two replisomes need not be physically connected for efficient DNA replication to occur [59], physical tethering of two CMGs has been suggested to play a role during sister chromatid cohesion by keeping the two ends of a growing replication bubble in close physical proximity [48]. Mechanistic studies on sister chromatid cohesion and parental histone redeposition at the replication fork are in their infancy, and present exciting new challenges for the field of chromosome replication.

## Replisome dynamics during fork progression

*In silico* reconstitution of double-hexamer formation using time-resolved cryo-EM has allowed for the visualisation of the structural intermediates that occur upon licensing of an origin of replication [22]. So far, however, structural analysis of all the molecular assemblies that co-ordinate downstream replication events have either been studied at equilibrium or as isolated subcomplexes stabilised with nucleotide analogues [33,36,60,61].

The precise nature of the molecular interactions between DNA polymerases and the rest of the core replisome is an important determinant in processes such as polymerase recycling and the mechanism of lagging-strand synthesis. Biochemical studies have revealed that the leading-strand polymerase Pol ε is tightly associated with the CMG helicase [34,41,62,63]. Conversely, the lagging-strand polymerase Pol δ is believed to be highly dynamic and physically disconnected from the replisome core [39]. Whether the DNA polymerases act dynamically or are stably tethered to the replisome core during fork progression remains to be established. A recent van Oijen and O'Donnell study utilised single-molecule fluorescence imaging to simultaneously monitor DNA synthesis and polymerase dynamics of individual replisomes in real time [64]. Reconstituted eukaryotic replisomes were found to be highly resistant to dilution, retaining the continuous presence of one Pol ε, one Pol δ and two Pol α molecules for the synthesis of tens of kilobases. However, all three polymerases were found to dynamically exchange into the replisome, when challenged with excess polymerases in solution (Figure 3B [64]). The kinetics of these observed polymerase exchange events were dependent on the concentration of polymerase molecules in solution. Notably, a single Pol δ molecule was observed to be reused for the synthesis of many Okazaki fragments, even when challenged with excess polymerases in solution. This apparent stability of Pol δ during lagging-strand synthesis is facilitated in part through its Pol32 subunit [64]. Indeed, Pol32 has previously been reported to interact with the Pol1 catalytic subunit of Pol α, suggesting a possible mechanism for Pol δ tethering to the replisome core [65]. Consistent with this scenario, cryo-EM analysis of Pol δ bound to primed-DNA reveals that the Pol32 subunit of Pol δ is surface-exposed and hence available to interact with other replisome factors.

The dynamics of the interactions between replisome components raise an immediate paradox. How can the replisome form a stable processive complex, while polymerases are easily exchanged? This same behaviour has been observed in other model systems and can be explained by the presence of multiple weak pair-wise protein–protein-binding sites linking polymerases with core replisome factors [66–69]. In this scenario, stable polymerase association is mediated by interaction with two or more simultaneous binding sites, as indeed observed in the CMG–Pol ε cryo-EM structure [34]. Under dilute conditions (or in the absence of a solution pool of proteins), complete polymerase dissociation would involve at least two steps. Transient disruption to one of the first interaction elements would be followed by rapid reassociation, preventing polymerase release. Things would change if competitor polymerase molecules were to be found in close proximity to these transiently vacated sites. Under these conditions, the polymerase exchange would be favoured as a transiently vacated interaction site would become occupied by a polymerase molecule recruited from the solution pool. Such a concentration-dependent multi-site exchange mechanism provides a general solution to tuning the stability of proteins at the replication fork to help handle obstacles and endogenous stressors during replication of large genomes.

We now understand that the Pol ε interaction with CMG is much more dynamic than originally suggested by the first cryo-EM imaging experiments, while Pol δ is emerging as a candidate component of the replisome core, in contrast with the textbook picture. Retention of Pol α and Pol δ over lagging-strand cycles has important implications for replisome coordination, presumably through the generation of priming, lagging-strand loops or a combination of both. Simultaneous observation of DNA looping and leading-strand synthesis during replication by T7 replisomes have demonstrated that most loop formation events occur during primer synthesis [70]. Coordination of DNA synthesis may be achieved through multiple reaction mechanisms involving the production of replication loops. Previous studies have implied the frequency to which possible mechanisms are utilised by the replisome is often guided by the physical connections between different proteins [71–73]. It remains to be established how lagging-strand primer hand-off, DNA polymerase activity and the formation of loops on the lagging strand are co-ordinated to achieve simultaneous synthesis of both strands in such a polymerase-tethered replisome. Understanding the molecular configurations available to the eukaryotic replisome during fork progression, especially during exchange events, will be exciting areas of the new investigation.

## Future directions

The ability to describe the compositional and conformational dynamics of the advancing replisome is an important new challenge for structural biologists. Recent biochemical reconstitution combined with single-particle cryo-EM techniques have provided important insights into the mechanism of DNA replication. At the same time, real-time single-molecule imaging of individual replisomes have started to reveal unexpected kinetic and compositional dynamics at the replication fork. Thus, the development of new structural tools will be required to characterise the molecular choreography that control and maintain the replisome during chromosome replication. Time-resolved imaging approaches, combined with *in silico* reconstitution techniques, have enabled the detection of sequential MCM helicase loading reactions on the minute timescale [22]. Other events leading to the replication fork establishment (e.g. Mcm10-triggered lagging-strand ejection from CMG) will likely require higher temporal resolution. Fortunately, the ability to isolate reaction intermediates using microfluidic devices coupled to spray-plunging technologies are rendering robust millisecond-resolution time-resolved cryo-EM a reality [74].

In parallel, computational methods to tackle complex structural flexibility and compositional heterogeneity are being developed. Three-dimensional classification strategies and multi-body refinement are established, powerful techniques that can describe non-discrete conformational heterogeneity in large molecular assemblies [75]. At the same time, new approaches are being developed to capture the full context of dynamic protein–DNA assemblies. Traditional single-particle approaches can report on the high-resolution structure of nucleoprotein complexes but often fail to report on the relative orientation of two particles spaced apart by a flexible DNA stretch. Protocols such as ReconSil [22] aim to overcome these technical limitations and map different protein-binding sites on the same DNA molecule. By positioning averaged DNA-bound structures back onto the original micrograph, overlaid to the corresponding raw particles, enhanced-signal views can be obtained for large DNA segments decorated by multiple proteins. This approach is powerful in describing complex replication reactions performed by compositionally dynamic protein assemblies. Combined with time-resolved resolved methods, these techniques enable observation of transient intermediates, which are lost during averaging when using traditional single-particle averaging methods. In summary, biochemical reconstitution combined with modern cryo-EM image processing promises to provide a complete understanding of the dynamic structural transitions that occur upon replication fork establishment and replisome progression.

## Perspectives

- *Importance of the field:* Studying chromosome replication is key to understanding genome stability. To understand the concerted function of multiple enzymes that form the eukaryotic replisome, we must describe their structural dynamics.
- *Current thinking:* Time-resolved cryo-EM methods allow us to establish the sequence of molecular events that drive replication origin transactions. Single-molecule approaches describe the unexpected dynamics of replisome components, posing new challenges to structural biology.
- *Future directions:* Development of time-resolved methods with millisecond resolution will allow access to key short-lived structural intermediates on the path to replication fork establishment. *In silico* reconstitution methods will enable understanding the concerted action of multiple replication enzymes on the broader context of replication fork progression.

## Abbreviations

cryo-EM: cryo-electron microscopy
ORC: Origin Recognition Complex

## Open Access

Open access for this article was enabled by the participation of The Francis Crick Institute in an all-inclusive *Read & Publish* pilot with Portland Press and the Biochemical Society under a transformative agreement with JISC.
